# Efficacy of typhoid conjugate vaccine: final analysis of a 4-year, phase 3, randomised controlled trial in Malawian children

**DOI:** 10.1016/S0140-6736(23)02031-7

**Published:** 2024-02-03

**Authors:** Priyanka D Patel, Yuanyuan Liang, James E Meiring, Nedson Chasweka, Pratiksha Patel, Theresa Misiri, Felistas Mwakiseghile, Richard Wachepa, Happy C Banda, Florence Shumba, Gift Kawalazira, Queen Dube, Nginache Nampota-Nkomba, Osward M Nyirenda, Tsion Girmay, Shrimati Datta, Leslie P Jamka, J Kathleen Tracy, Matthew B Laurens, Robert S Heyderman, Kathleen M Neuzil, Melita A Gordon, TyVAC team, Priyanka D Patel, Yuanyuan Liang, James E Meiring, Nedson Chasweka, Pratiksha Patel, Theresa Misiri, Felistas Mwakiseghile, Richard Wachepa, Happy C Banda, Florence Shumba, Gift Kawalazira, Queen Dube, Nginache Nampota-Nkomba, Osward M Nyirenda, Tsion Girmay, Shrimati Datta, Leslie P Jamka, J Kathleen Tracy, Matthew B Laurens, Robert S Heyderman, Kathleen M Neuzil, Melita A Gordon, Chrissy Banda, David Banda, Josephine Chilongo, Amisa Chisale, Mark Haward, Harrison Msuku, John Ndaferankhande, Chancy Nyirongo, Patricia Phula, James Tamani, Victoria Mapemba, Fleesie Hubbard, Melissa Myers, Tamar Pair

**Affiliations:** a Malawi-Liverpool-Wellcome Program, Kamuzu University of Health Sciences, Blantyre, Malawi; b Department of Epidemiology and Public Health, University of Maryland School of Medicine, Baltimore, MD, USA; c Center for Vaccine Development and Global Health, University of Maryland School of Medicine, Baltimore, MD, USA; d Department of Infection, Immunity and Cardiovascular Disease, University of Sheffield, Sheffield, UK; e District Health Office, Blantyre District Council, Blantyre, Malawi; f Ministry of Health, Blantyre, Malawi; g Blantyre Malaria Project, Kamuzu University of Health Sciences, Blantyre, Malawi; h Division of Infection and Immunity, University College London, London, UK

## Abstract

**Background:**

Randomised controlled trials of typhoid conjugate vaccines among children in Africa and Asia have shown high short-term efficacy. Data on the durability of protection beyond 2 years are sparse. We present the final analysis of a randomised controlled trial in Malawi, encompassing more than 4 years of follow-up, with the aim of investigating vaccine efficacy over time and by age group.

**Methods:**

In this phase 3, double-blind, randomised controlled efficacy trial in Blantyre, Malawi, healthy children aged 9 months to 12 years were randomly assigned (1:1) by an unmasked statistician to receive a single dose of Vi polysaccharide conjugated to tetanus toxoid vaccine (Vi-TT) or meningococcal capsular group A conjugate (MenA) vaccine. Children had to have no previous history of typhoid vaccination and reside in the study areas for inclusion and were recruited from government schools and health centres. Participants, their parents or guardians, and the study team were masked to vaccine allocation. Nurses administering vaccines were unmasked. We did surveillance for febrile illness from vaccination until follow-up completion. The primary outcome was first occurrence of blood culture-confirmed typhoid fever. Eligible children who were randomly assigned and vaccinated were included in the intention-to-treat analyses. This trial is registered at ClinicalTrials.gov, NCT03299426.

**Findings:**

Between Feb 21, 2018, and Sept 27, 2018, 28 130 children were vaccinated; 14 069 were assigned to receive Vi-TT and 14 061 to receive MenA. After a median follow-up of 4·3 years (IQR 4·2–4·5), 24 (39·7 cases per 100 000 person-years) children in the Vi-TT group and 110 (182·7 cases per 100 000 person-years) children in the MenA group were diagnosed with a first episode of blood culture-confirmed typhoid fever. In the intention-to-treat population, efficacy of Vi-TT was 78·3% (95% CI 66·3–86·1), and 163 (129–222) children needed to be vaccinated to prevent one case. Efficacies by age group were 70·6% (6·4–93·0) for children aged 9 months to 2 years; 79·6% (45·8–93·9) for children aged 2–4 years; and 79·3% (63·5–89·0) for children aged 5–12 years.

**Interpretation:**

A single dose of Vi-TT is durably efficacious for at least 4 years among children aged 9 months to 12 years and shows efficacy in all age groups, including children younger than 2 years. These results support current WHO recommendations in typhoid-endemic areas for mass campaigns among children aged 9 months to 15 years, followed by routine introduction in the first 2 years of life.

**Funding:**

Bill & Melinda Gates Foundation.

## Introduction

Typhoid fever imposes a high burden of disease and an increasing threat to public health in sub-Saharan Africa, Asia, and Oceania. The scarcity of diagnostic tools and surveillance platforms makes estimating the burden of *Salmonella enterica* serotype Typhi (*S* Typhi) challenging and, as a result, typhoid fever incidence is underestimated in many low-income settings.^1^, ^2^, ^3^, ^4^ The case fatality rate for typhoid fever is estimated to be 1% in Asia, rising to 5·4% in Africa,^5^ and is highest when treatment and access to care is delayed or inadequate. Severe and complicated disease, which is estimated to comprise 26% of cases, is associated with substantial morbidity.^5^ The global emergence of antibiotic resistance, including multidrug resistance against first-line agents (ie, ampicillin, chloramphenicol, and cotrimoxazole) and fluoroquinolone non-susceptibility, has contributed to transcontinental spread of typhoid fever, and reduces the efficacy of existing treatments.^6^, ^7^, ^8^, ^9^ The rise of extensively drug-resistant typhoid strains in Asia further threatens public health, heightening the importance of introducing typhoid vaccination in endemic regions.^10^, ^11^

Before 2017, globally available vaccines against typhoid fever included a parenteral Vi polysaccharide vaccine (Vi-PS) and the live oral Ty21a vaccine. Neither, however, are approved for use in children younger than 2 years, and both require repeated dosing for durable efficacy. A Cochrane systematic review estimated that the efficacy of Vi-PS fell from 69% (95% CI 63–74) after 1 year to 55% (95% CI 30–70) after 3 years.^12^ The vaccine efficacy of Ty21a was estimated to be 50% (95% CI 35–61) after 2·5–3 years. As a result, although these vaccines are used for travellers from high-income settings and for outbreak control, and although they are recommended by WHO,^13^ they have not been used in routine immunisation programmes in low-income and middle-income countries.

**Research in context**

**Evidence before this study**

Until 2018, the only WHO-recommended vaccines available were Vi polysaccharide or Ty21a live oral vaccines, which were not suitable for children younger than 2 years and were accordingly rarely used in low-income settings. WHO recommended typhoid conjugate vaccine (TCV) in 2018 for mass catch-up campaigns for those up to 15 years of age and routine introduction at age 9–15 months in endemic countries, with priority to countries with high typhoid incidence or high antimicrobial resistance among *S* Typhi isolates. Randomised controlled trials in Nepal, Bangladesh, and Malawi established the efficacy of the vaccine at 18–36 months after vaccination.

We searched PubMed and the Cochrane Central Register of Controlled Trials for clinical trials involving children using the terms “typhoid conjugate vaccine” and “efficacy” between Jan 1, 1970, and March 29, 2023, with no language restrictions and filters for the following age ranges: 1–23 months, 2–5 years, 6–12 years, and 13–18 years. Five clinical trials evaluating the efficacy of TCV in children in Malawi, Nepal, Bangladesh, Viet Nam, and India were identified. The TCV efficacy trials conducted in Malawi, Nepal, and Bangladesh used single-dose Vi-TT and reported vaccine efficacy against blood culture-confirmed typhoid fever of 80·7% (95% CI 64·2–89·6) at 18–24 months in Malawi, 79·0% (61·9–88·5) at 24 months in Nepal, and 85% (97·5% CI 76–91) at 18 months in Bangladesh.

The other two trials identified in the search used two-dose regimens of Vi polysaccharide conjugated to Pseudomonas aeruginosa exotoxin A (Vi-rEPA; in Viet Nam) and Vi-tetanus toxoid conjugated typhoid vaccine (PedaTyph; in India). The trial of Vi-rEPA began in 1998 and found a vaccine efficacy of 91·5% (95% CI 77·1–96·6) against blood culture-confirmed typhoid fever through 27 months of follow-up in children aged 2–5 years. After unmasking, passive surveillance was conducted for another 19 months, with a vaccine efficacy of 82·4% (22·3–99·1) during that period. The Vi-rEPA did not progress further in development and was not marketed. The PedaTyph trial reported a vaccine efficacy of 100% (97·6–100) for a two-dose regimen over a 12-month surveillance period in children aged 6 months to 12 years. In addition to the short follow-up, this trial enrolled a small sample size, was not individually randomised, and did not include a control vaccine.

**Added value of this study**

This is the first randomised, controlled, double-blind trial to evaluate the longer-term efficacy of single-dose TCV in a typhoid fever-endemic setting from 9 months of age. Our study provides evidence that Vi-TT provides durable overall protection beyond 48 months after vaccination among children vaccinated between 9 months and 12 years of age, with little decline in efficacy over time. We estimated that vaccine efficacy reduced over time by only 1·3% per year over 4 years. The longer durability of protection translates into a lower number needed to vaccinate to prevent each case of blood culture-confirmed typhoid fever. An age-stratified analysis found that the vaccine is efficacious in all age groups, including children younger than 2 years old.

**Implications of all the available evidence**

Our data support robust and durable overall protection—for at least 4 years—in children vaccinated according to the WHO recommendation of a single dose of TCV for infants and children in typhoid-endemic areas. The results from this longer term trial support the high estimated cost-effectiveness of these vaccines, generated using assumptions that were based on previous trials of shorter duration. Further long-term data to assess the durability of protection, particularly in young children following WHO-recommended routine introduction of TCV, are warranted.

In endemic areas, typhoid fever occurs over a broad age range, from the first year of life into adulthood, with incidence peaking in most endemic settings among children aged 5–15 years.^14^ WHO prequalification of a typhoid conjugate vaccine (TCV) comprising a Vi polysaccharide conjugated to tetanus toxoid (Vi-TT, Typbar TCV, Bharat Biotech International) in 2017, and a TCV comprising a Vi polysaccharide conjugated to a variant of diphtheria toxin (Vi-CRM197, TYPHIBEV, Biological E) in 2020, offers a major opportunity to address the substantial burden of typhoid fever with vaccines more suitable for use in endemic settings. TCVs can be administered to children younger than 2 years of age and induce more durable immunity than previous vaccines, enhancing their suitability.^15^ WHO has recommended single-dose TCV be incorporated into the Expanded Program on Immunization (EPI) at 9 months or 15 months of age. For more immediate effect, WHO recommends a one-time single-dose mass catch-up campaign in children up to 15 years of age, with priority given to countries with a high burden of disease or antimicrobial resistant *S* Typhi.^16^ Modelling studies have confirmed the cost-effectiveness of these combined strategies in high-incidence countries.^17^

Three large trials of Vi-TT were conducted beginning in 2017 among children aged 9 months to 12–15 years in Nepal,^18^ Malawi,^19^ and Bangladesh.^20^ These trials cumulatively enrolled more than 100 000 children and demonstrated the tolerability and safety of single-dose Vi-TT, with efficacy ranging from 79–85% against blood culture-confirmed typhoid fever, for follow-up periods ranging between 18 months and 36 months.^21^ The vaccine efficacy was remarkably consistent across these different epidemiological settings, in both individually randomised and cluster randomised study designs.^18^, ^19^, ^20^ These safety and efficacy data have already informed the introduction of TCVs through routine immunisation and mass campaigns for children from 6 months to 15 years of age in several typhoid-endemic countries.^22^

In this randomised controlled trial in Malawi that enrolled more than 28 000 children, we previously reported that Vi-TT was safe and well-tolerated, with an efficacy of 80·7% (95% CI 64·2–89·6) against first episode, blood culture-confirmed typhoid fever after 18–24 months of follow-up.^19^ Study surveillance was temporarily halted due to the COVID-19 pandemic but had not accrued sufficient cases at that time to report on efficacy in children younger than 2 years of age. Passive surveillance resumed after a 21-week COVID-19 hiatus and was continued until the prespecified trial end date of Sept 30, 2022. Herein, we present the final efficacy results of single-dose Vi-TT over an extended median follow-up time of 4·3 years, and by age group, including children younger than 2 years of age.

## Method

### Study design

This phase 3, double-blind, randomised controlled efficacy trial was conducted in the Ndirande and Zingwangwa urban townships of Blantyre, Malawi.^19^, ^23^ Children were randomly assigned to receive one of two vaccines, and followed up until the prespecified end-date of the trial. The trial had four passive surveillance sites: three primary health-care facilities (Ndirande health centre, Zingwangwa health centre, and Gateway clinic) and one tertiary referral hospital (Queen Elizabeth Central Hospital). Details of the study design have been previously published.^23^ Local ethics approval was obtained from the Malawi National Health and Sciences Research Committee, and international ethics approval from the University of Liverpool Ethics Review Board and the University of Maryland Baltimore Institutional Review Board. Results and safety were regularly reviewed by a Data and Safety Monitoring Board. This study was conducted under Good Clinical Practice guidelines, with internal and external monitoring for quality.

### Participants

Children were recruited from 20 government schools and two health centres in the study area and were enrolled by study clinicians. Inclusion criteria required that children were aged 9 months to 12 years, resided in the study areas, had no previous history of typhoid vaccination, and were free of acute illness. Community engagement to inform about the study happened at all stages of the trial using appropriate and varied methods, as described elsewhere.^24^ Written informed consent was obtained from the parent or guardian of all children before any study procedures, and written assent was also required for all children aged 8 years and older.^19^

### Randomisation and masking

Children were randomly assigned (1:1) to receive a single dose of either Vi-TT (Bharat Biotech International, Hyderabad, India) or a meningococcal capsular group A conjugate control vaccine (MenA, Serum Institute of India, Pune, India). MenA was chosen as the comparator because MenA is WHO-prequalified, and, like Vi-TT, is a single-dose polysaccharide-conjugate vaccine approved for the age groups enrolled. Block randomisation was used with block sizes of 6–12. The randomisation allocation sequence was generated by an unmasked statistician using the blockrand package (version 1.3) in R (version 3.4.1). The randomisation allocation sequence was uploaded to password-protected tablets and accessed only by a restricted number of unmasked vaccination nurses in real time, immediately before vaccination, in screened-off vaccination areas. Masking was monitored in real-time by senior study team members who supervised the general flow and process from outside the blinded vaccination area. Participants, their parents or guardians, and the study team involved in subsequent disease surveillance screening, eligibility assessment, and follow-up were masked to vaccine allocation.

### Procedures

MenA and Vi-TT were administered intramuscularly.^19^ Children aged 9–11 months had the trial vaccine co-administered with measles–rubella vaccine as part of their routine Malawi EPI schedule.^25^

The passive surveillance sites were the primary destinations for children with fever seeking medical attention in the study areas. At the time of vaccination, parents and guardians were informed to bring their children to any of the passive surveillance sites if they had a fever. This message was reinforced through post-vaccination community engagement throughout the trial period.

Vaccinated participants who presented to one of the four passive surveillance facilities were screened for eligibility for blood culture collection per the protocol. Children who met the prespecified case definition (ie, measured axillary temperature of ≥38°C; history of documented or subjective fever for 3 days or more; or hospital admission with fever of any duration) had blood-culture and malaria rapid diagnostic tests performed. Clinical details and previous antibiotic use by the participants were recorded on an electronic case report form by study team clinicians. Immediate clinical management was administered according to local guidelines by the attending clinician, and participants whose blood cultures isolated *S* Typhi were contacted to ensure that they were started on an appropriate antibiotic with reference to the antibiotic sensitivity report. Blood culture and microbiology methods were as previously described.^19^ Individuals with blood culture-confirmed typhoid were prospectively followed up once every 2 weeks until their typhoid illness resolved. Participants with blood cultures that isolated pathogens other than *S* Typhi were contacted to ensure they were started on treatment as per Malawi's standard treatment guidelines.

This trial was originally designed to have a minimum follow-up period of 24 months per participant; however, Malawi experienced four waves of COVID-19 during the trial, and the study was paused on April 3, 2020, due to COVID-19 restrictions in the country.^26^ Results at this point (after 18–24 months of follow-up) were analysed and published, without individual unmasking, since the predetermined minimum number of total cases across all age groups had been attained.^19^ The COVID-19 suspension ended on Aug 27, 2020, and disease surveillance resumed, following all COVID-19 mitigation strategies as per Malawi's national guidelines.^26^ The protocol was modified twice to extend the final analysis date first to Sept 30, 2021, and then to Sept 30, 2022. Passive surveillance therefore continued until Sept 30, 2022, by which time all participants had a minimum follow-up period of 48 months.

### Outcomes

The primary outcome was the occurrence of blood culture-confirmed typhoid fever. Secondary outcomes included blood culture-confirmed typhoid fever occurring at least 14 days after vaccination and safety outcomes. Safety data (solicited and unsolicited adverse events, and serious adverse events) were prospectively recorded, per protocol. Any occurrence that resulted in hospital admission, prolonged hospital stay, life-threatening condition, persistent or significant disability, or death was reported as a serious adverse event. All serious adverse events within 18 months of vaccination were reported to the relevant ethics committees within 24 h of awareness.

### Statistical analysis

All trial data were collected directly on tablets by study staff and uploaded to a REDCap database. Data were carefully reviewed and incomplete, incorrect, or inaccurate data were identified and modified through site queries and additional investigation. When this process was complete, the database was locked. Details of sample size and power calculations have been reported.^23^ Briefly, assuming 75% vaccine efficacy, the minimum number of total cases needed to test the null hypothesis that the vaccine has no protective efficacy (ie, vaccine efficacy ≤0), with 90% power, was 30. The primary analysis was conducted under the intention-to-treat principle, in which all children who were randomly assigned and received a dose of a vaccine were included in the analysis; vaccine group was defined based on the vaccine that was randomly assigned not the vaccine that was actually received. In the intention-to-treat analysis, all first occurrences of blood culture-confirmed typhoid cases after vaccination were used. In the per-protocol analysis, only children who received the assigned vaccine and completed the trial without any protocol deviations were included. All occurrences of blood culture-confirmed typhoid cases within 14 days after vaccination were excluded in the per-protocol analysis.

For each child, the follow-up time since the date of vaccination was calculated as the smallest of the following: time to the first episode of typhoid fever; time to withdrawal from the trial, loss to follow-up, death, or confirmed relocation out of the trial area; or the time to the end of the analysis period (Sept 30, 2022). Typhoid incidence rate was calculated as the number of first episodes of blood culture-confirmed typhoid fever divided by the total follow-up time. The vaccine efficacy was calculated as (1 – incidence rate ratio) × 100%, where the incidence rate ratio was defined as the ratio of the incidence rate in the Vi-TT group to that in the MenA group. The absolute risk reduction was calculated as the risk of blood culture-confirmed typhoid fever in the MenA group minus that in the Vi-TT group. The number needed to vaccinate to prevent one case of blood culture-confirmed typhoid fever was calculated as 1 divided by the absolute risk reduction.

Subgroup analyses were conducted to evaluate the incidence rate of blood culture-confirmed typhoid fever, incidence rate ratio, and vaccine efficacy among the following prespecified subgroups: age at the time of vaccination (ie, <2 years; 2–4 years; ≥5 years), sex, and study site residence (ie, Ndirande or Zingwangwa). The test of homogeneity in incidence rate ratio and vaccine efficacy across subgroups was conducted using the Mantel-Haenszel method.

We used the Kaplan-Meier method to estimate the cumulative incidence of blood culture-confirmed typhoid fever since the date of vaccination for each vaccine group with p values calculated using both a log-rank test and Wilcoxon–Breslow–Gehan test. The incidence rate for each vaccine group and the vaccine efficacy were computed for various time intervals: cumulative at 1 year, 2 years, 3 years, 4 years, and up to 4·61 years (the longest follow-up observed up to the prespecified end-date) after vaccination; and in discrete time periods, 0–1 years, 1–2 years, 2–3 years, 3–4 years, and 4–4·61 years after vaccination. The trend in vaccine efficacy over the discrete intervals was examined using a weighted linear regression. All analyses were performed using Stata/SE version 17.

This trial is registered at ClinicalTrials.gov, NCT03299426.

### Role of the funding source

The funder of the study had no role in study design, data collection, data analysis, data interpretation, or writing of the report.

## Results

**Figure 1:**
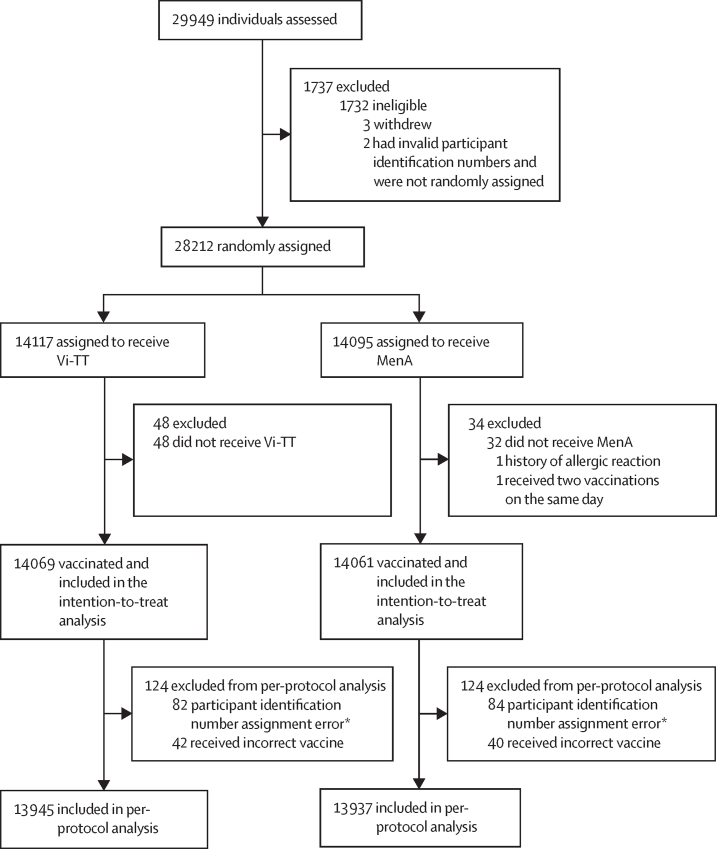
Trial profile 248 participants were excluded from the per-protocol analysis due to a participant identification number assignment error or vaccine administration error. MenA=meningococcal capsular group A conjugate vaccine. Vi-TT=Vi polysaccharide tetanus toxoid typhoid conjugate vaccine. *166 pairs of children received the same participant identification number due to a duplicate bar code printing error. The second participant who received the duplicate number within a pair was assigned a new number and excluded from the per-protocol analysis.

A detailed enrolment and randomisation flow chart has been published previously.^19^ Briefly, between Feb 21, 2018, and Sept 27, 2018, 29 949 healthy children were screened for inclusion, a total of 28 217 were eligible, and 28 130 participants were vaccinated. Five children were eligible, but not randomly assigned to treatment groups as three parents or guardians withdrew their child from the study between eligibility assessment and randomisation, and two did not have valid participant identification numbers, due to a tablet scanning error. 28 130 participants were included in the intention-to-treat analysis and 27 882 in the per-protocol analysis (figure 1).^19^ The participants' median age was 6·0 years (range 0·8–12·0) in the intention-to-treat population and baseline characteristics did not differ between vaccine groups (table 1).^19^

**Table 1:** Baseline characteristics of the intention-to-treat population

		**Vi-TT (n=14 069)**	**MenA (n=14 061)**
Age at enrolment (years)			
	Mean (SD)	6·1 (3·3)	6·2 (3·3)
	Median (range)	6·0 (0·8–12·0)	6·0 (0·8–12·0)
Age group			
	<2 years	1555 (11·1%)	1600 (11·4%)
	2–4 years	3503 (24·9%)	3579 (25·5%)
	≥5 years	9011 (64·1%)	8882 (63·2%)
Sex			
	Female	7065 (50·2%)	7231 (51·4%)
	Male	7004 (49·8%)	6830 (48·6%)
Ethnicity			
	Black African	14 069 (100·0%)	14 061 (100·0%)
Study site			
	Ndirande	8863 (63·0%)	8832 (62·8%)
	Zingwangwa	5206 (37·0%)	5229 (37·2%)

Data are n (%) unless otherwise specified. Vi-TT=Vi polysaccharide tetanus toxoid typhoid conjugate vaccine. MenA=meningococcal capsular group A conjugate vaccine.

**Figure 2:**
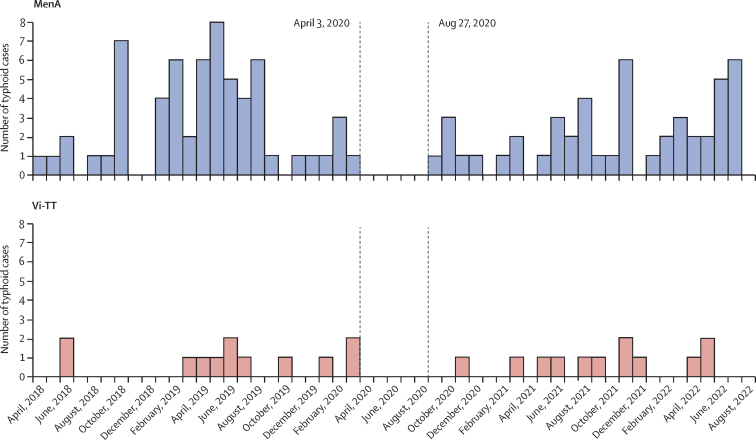
Number of blood-culture positive typhoid cases in the intention-to-treat population, by date and vaccine group Dates of COVID-19 surveillance interruptions are shown with dotted vertical lines. MenA=meningococcal capsular group A conjugate vaccine. Vi-TT=Vi polysaccharide tetanus toxoid typhoid conjugate vaccine.

Between Feb 21, 2018, and Sept 30, 2022, 39 174 study health facility visits by 15 371 study participants were assessed for eligibility for blood culture collection. Of these, 10 777 (28%) visits met criteria for blood-culture collection, and 10 136 (94% of eligible) blood cultures were collected from 7251 individual participants. Among these blood cultures, 136 (1·3%) grew *S* Typhi. Among the 136 blood culture-confirmed typhoid infections, two participants contracted typhoid twice. Only the first episode was included in the intention-to-treat analysis as per the statistical analysis plan, resulting in a total of 134 blood culture-confirmed typhoid cases in the intention-to-treat analysis (figure 2). Among the 134 cases of typhoid, three occurred within 14 days after vaccination and were therefore excluded from the per-protocol analysis, resulting in a total of 131 blood culture-confirmed typhoid cases in the per-protocol analysis.

Passive surveillance activities were paused in compliance with national mandates due to COVID-19. Disruption on blood cultures taken and detection of culture-confirmed typhoid infections caused a complete cessation of activity from April 3, 2020, to Aug 26, 2020, followed by gradual recovery of both case detection and blood culture sampling (figure 2, appendix p 1). Of note, sampling and case detection numbers had substantially recovered in the final 12 months of the trial. No discernible annual seasonality effect was observed for typhoid infection.

Overall vaccine efficacy in preventing a first episode of blood culture-confirmed typhoid fever for the entire study period was 78·3% (95% CI 66·3–86·1) in the intention-to-treat analysis, with 24 (0·2%) of 14 069 individuals contracting typhoid in the Vi-TT arm (39·7 per 100 000 person-years) and 110 (0·8 %) of 14 061 individuals in MenA arm (182·7 per 100 000 person-years; table 2). The median follow-up time was 4·3 years (IQR 4·2–4·5). The absolute risk reduction was 6·1 typhoid infections per 1000 vaccinated children, corresponding to a number needed to vaccinate of 163 (95% CI 129–222) to prevent one case of typhoid fever. The vaccine efficacy in the per-protocol analysis was 80% (68·3–87·3), with 22 (0·2%) of 13 945 blood culture-confirmed typhoid cases in the Vi-TT arm (36·7 cases per 100 000 person-years) and 109 (0·8%) of 13 937 blood culture-confirmed typhoid cases in the MenA arm (182·7 per 100 000 person-years), similar to the intention-to-treat results (table 2).

**Table 2:** Blood-culture-confirmed typhoid fever and vaccine efficacy

		**Number at risk**	**Total follow-up time (person-years)**	**Number of cases of blood-culture-confirmed typhoid fever**	**Incidence rate (per 100 000 person-years; 95% CI)**	**Protective efficacy of Vi-TT (95% CI)**	Absolute risk reduction per 1000 children (95% CI)*	**Number needed to vaccinate (95% CI)**†
**Intention-to-treat population**								
Vi-TT		14 069	60 500	24	39·7 (25·4–59·0)	78·3% (66·3–86·1)	6·1 (4·5–7·7)	163 (129–222)
MenA		14 061	60 220	110	182·7 (150·1–220·2)	Ref	Ref	Ref
**Age at vaccination**								
<2 years								
	Vi-TT	1555	6586	4	60·7 (22·8–161·8)	70·6% (6·4–93·0)	6·2 (1·0–11·4)	162 (88–1035)
	MenA	1600	6773	14	206·7 (122·4–349·0)	Ref	Ref	Ref
2–4 years								
	Vi-TT	3503	15 007	5	33·3 (13·9–80·1)	79·6% (45·8–93·9)	5·6 (2·6–8·6)	180 (117–391)
	MenA	3579	15 297	25	163·4 (110·4–241·9)	Ref	Ref	Ref
≥5 years								
	Vi-TT	9011	38 907	15	38·6 (23·2–64·0)	79·3% (63·5–89·0)	6·3 (4·3–8·4)	158 (120–233)
	MenA	8882	38 151	71	186·1 (147·5–234·8)	Ref	Ref	
**Per-protocol population**								
Vi-TT		13 945	59 942	22	36·7 (23·0–55·6)	80·0% (68·3–87·3)	6·2 (4·6–7·8)	160 (127–216)
MenA		13 937	59 662	109	182·7 (150·0–220·4)	Ref	Ref	Ref

Vi-TT=Vi polysaccharide tetanus toxoid typhoid conjugate vaccine. MenA=meningococcal capsular group A conjugate vaccine.

* Absolute risk reduction (risk in the MenA group minus risk in the Vi-TT group) is the total reduction in the risk of blood-culture-confirmed typhoid fever that resulted from vaccination with TCV.

† Number needed to vaccinate is the number of children that would need to be vaccinated to prevent one case of blood-culture-confirmed typhoid fever.

The estimated cumulative vaccine efficacy was 83·4% after 1 year, 80·7% after 2 years, 80·1% after 3 years, 77·1% after 4 years, and 78·3% after 4·61 years (the longest follow-up observed at the prespecified study end date) following vaccination (table 3). Considered as discrete increments, the efficacy was 83·4% at 0–1 years, 77·0% at 1–2 years, 77·0% at 2–3 years, 68·2% at 3–4 years, and 90·1% at 4–4·61 years (table 3; appendix p 3). The trend in efficacy by yearly increments, analysed using a random effects meta-regression taking into account the precision for each annual estimate, suggests there was a 1·3% decline in vaccine efficacy each year (slope –0·013, 95% CI –0·098 to 0·072, p=0·77, appendix p 4).

**Table 3:** Blood-culture-confirmed typhoid fever and vaccine efficacy over time in the intention-to-treat population

		**Number at risk**	**Total follow-up time (person-years)**	**Number of cases of blood-culture-confirmed typhoid fever**	**Incidence rate (per 100 000 person-years; 95% CI)**	**Protective efficacy of Vi-TT (95% CI)**
**Cumulative time since vaccination**						
0–1 years						
	Vi-TT	14 069	14 058	6	42·7 (19·2–95)	83·4% (60·1–94·3)
	MenA	14 061	14 036	36	256·5 (185·0–355·6)	Ref
0–2 years						
	Vi-TT	14 069	28 104	12	42·7 (24·2–75·2)	80·7% (63·8–90·5)
	MenA	14 061	28 021	62	221·3 (172·5–283·8)	Ref
0–3 years						
	Vi-TT	14 069	42 135	15	35·6 (21·5–59·1)	80·1% (65·0–89·4)
	MenA	14 061	41 983	75	178·6 (142·5–224·0)	Ref
0–4 years						
	Vi-TT	14 069	56 121	23	41·0 (27·2–61·7)	77·1% (63·7–86·1)
	MenA	14 061	55 889	100	178·9 (147·1–217·7)	Ref
0–4·61 years						
	Vi-TT	14 069	60 500	24	39·7 (25·4–59·0)	78·3% (66·3–86·1)
	MenA	14 061	60 220	110	182·7 (150·1–220·2)	Ref
**Discrete time since vaccination**						
First year						
	Vi-TT	14 069	14 058	6	42·7 (19·2–95·0)	83·4% (60·1–94·3)
	MenA	14 061	14 036	36	256·5 (185·0–355·6)	Ref
Second year						
	Vi-TT	14 050	14 046	6	42·7 (19·2–95·1)	77·0% (42·9–92·3)
	MenA	14 006	13 985	26	185·9 (126·6–273·1)	Ref
Third year						
	Vi-TT	14 043	14 031	3	93·1 (54·1–160·3)	77·0% (16·4–95·8)
	MenA	13 976	13 963	13	93·1 (54·1–160·3)	Ref
Fourth year						
	Vi-TT	13 996	13 986	8	57·2 (28·6–114·4)	68·2% (27·2–87·6)
	MenA	13 928	13 906	25	179·8 (121·5–266·1)	Ref
Fifth year (up to 4·61 years)						
	Vi-TT	13 980	4379	1	22·8 (3·2–162·1)	90·1% (30·5–99·8)
	MenA	13 893	4331	10	230·9 (124·2–429·1)	Ref

Vi-TT=Vi polysaccharide tetanus toxoid typhoid conjugate vaccine. MenA=meningococcal capsular group A conjugate vaccine.

**Figure 3:**
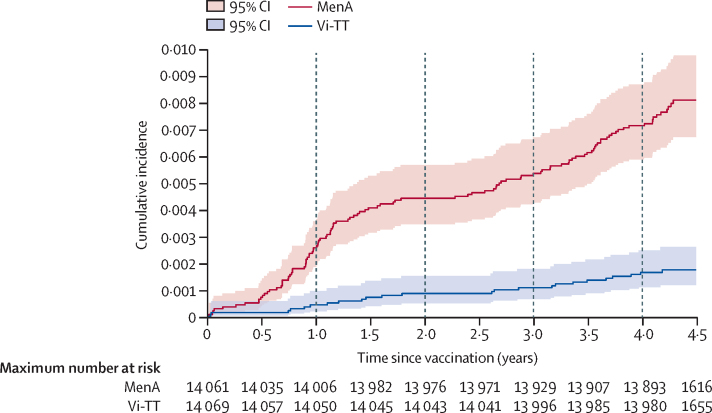
Kaplan-Meier estimates of the cumulative incidence of blood-culture positive typhoid fever Curves begin on vaccination day 0 and are for the intention-to-treat population by vaccine group. MenA=meningococcal capsular group A conjugate vaccine. Vi-TT=Vi polysaccharide tetanus toxoid typhoid conjugate vaccine.

In the intention-to-treat analysis, Kaplan-Meier survival curves showed a consistent and progressive separation in the cumulative incidence between the Vi-TT and MenA groups (p<0·0001; figure 3), confirming ongoing vaccine efficacy throughout the duration of the trial. This result is seen despite some flattening of the pattern of cumulative incidence around year 2, due to COVID-19 disruption (appendix p 2).

Considering the results by age, the incidence of blood culture-confirmed typhoid fever in the intention-to-treat population for the MenA group was high in all age-groups, with 206·7 per 100 000 person-years for children younger than 2 years, 163·4 for children aged 2–4 years, and 186·1 for children aged 5–12 years (table 2). The overall vaccine efficacy was similar among the three age groups (70·6% for children younger than 2 years, 79·6% for children aged 2–4 years, and 79·3% for children aged 5–12 years; p=0·85). The number needed to vaccinate was also similar across all age groups. Vaccine efficacy was not significantly different by sex (82%, 95% CI 64·3–91·8 among males and 74·5%, 53·6–86·9 among females; p=0·44), nor by study site residence (76·4%, 58–87·5 in Ndirande and 80·9%, 60·5–91·8 in Zingwangwa; p=0·65; appendix p 1).

During the duration of the trial, there were 15 hospital admissions due to typhoid fever in the MenA group, and six hospital admissions due to typhoid fever in the Vi-TT group. There was one death from typhoid fever in a child who received the MenA vaccine. Secondary endpoint safety results per protocol (solicited and unsolicited adverse events to 30 min, 7 days, and 28 days; serious adverse events to 28 days and 6 months) have been reported previously.^19^, ^25^ There were no serious adverse events or deaths attributable to the vaccine at any point in the trial.

*S* Typhi isolates from 135 (99%) of the 136 typhoid infections were multidrug resistant (ie, resistant to cotrimoxazole, chloramphenicol, and ampicillin) and isolates from six (4·4%) infections were also non-susceptible to fluoroquinolone. One isolate was fully susceptible to all antimicrobials tested. No extensively drug resistant (ie, resistant to cotrimoxazole, chloramphenicol and ampicillin, fluoroquinolone and third-generation cephalosporins) *S* Typhi isolates were detected.

## Discussion

In this large, randomised controlled trial among Malawian children aged 9 months to 12 years at vaccination, a single dose of Vi-TT offered at least 4 years of protection against blood culture-confirmed typhoid fever, with a cumulative vaccine efficacy of 78·3%. The loss of vaccine efficacy over time, across all age groups, was estimated to be only 1·3% per annum over more than 4 years. By showing durable protection, this study further highlights the probable public health benefit and economic value of TCVs. Longer follow-up until at least 48 months has identified an increased number of cases prevented by the original single-dose vaccine intervention. The estimated number needed to vaccinate to prevent one case of typhoid is based on the reduction in absolute risk of typhoid in individuals vaccinated with Vi-TT compared with those vaccinated with MenA. Our data show that the estimated number needed to vaccinate is now 163 children, which is substantially lower than the previous estimate of 278 children reported at the 18–24-month analysis, due to the additional infections prevented with the longer duration of follow-up. These results are likely to be reflected in more beneficial cost-effectiveness analyses, representing enhanced value for policy makers.

We found protection in all age groups, including children younger than 2 years of age. Durability of protection in those younger than 2 years is particularly important, given our findings that the incidence of typhoid fever in the control group is similar across age-bands. Therefore, the WHO-recommended single-dose TCV administered in the first or second year of life will need to protect a child over many years of risk. Our vaccine efficacy point estimate of 70·6% in this youngest age group is similar to the total vaccine effectiveness point estimate of 81% reported for Vi-TT in children younger than 2 years of age in a cluster-randomised trial in Bangladesh.^20^ The slightly higher point estimate in Bangladesh can be attributed to the shorter duration of follow-up (17 months in the Bangladesh study versus 4·3 years in this study), and the potential for indirect effects in the cluster-randomised study design.

Although the results of this study are strongly supportive of current WHO recommendations, continued data are needed from real-world post-introduction effectiveness and impact studies, to assess the strength and durability of protection, particularly in those children who receive vaccine during the routine EPI visit. Although the point estimates of vaccine efficacy in our study did not differ significantly by age group, the lowest point estimate was seen in the youngest children. This pattern is similar to that reported from an individually randomised efficacy trial in Nepal, where children younger than 5 years had a lower point estimate of vaccine efficacy for Vi-TT than children aged 5 years and older.^18^, ^19^ Insufficient numbers of children were enrolled in the Nepal study before 2 years of age to assess efficacy in that age stratum. Further, immunogenicity data from both the Nepal and this trial showed numerically more rapid decline in vaccine-induced antibody titres in younger children compared with older age groups but this did not reach statistical significance.^18^, ^25^ A mathematical model of the transmission dynamics of *S* Typhi predicted that a waning of vaccine-induced immunity could lead to a rebound in typhoid incidence 5–15 years after vaccine introduction, emphasising the need for longer term efficacy data.^27^ Whether additional benefit could be gained with a second dose of vaccine is unknown. An immunogenicity study of a booster dose of Vi-TT administered approximately 5 years after the initial dose of Vi-TT among a subset of the youngest children who were enrolled in this study is currently underway in Malawi.

This trial had some limitations. The number of children presenting for passive surveillance and blood culture collection decreased during the first 2 years. Any such cohort trial conducted over more than 4 years in an urban African setting would be expected to have some loss to follow-up or declining surveillance attendance due to migration out of the study area and increasing age of the cohort. Although this decline could result in underestimation of the true incidence of disease, it would not be expected to cause bias or affect the vaccine efficacy result, as these factors would be unrelated to vaccine allocation groups, for which participants and their parents or guardians were masked. The under-estimation of true disease incidence, however, would result in a number needed to vaccinate that is lower than calculated herein, meaning that the probable health and economic benefits of vaccination would be even greater than identified from the data presented here.

The COVID-19 pandemic further disrupted surveillance activities, with an initial suspension of study activities for nearly 5 months, followed by resumption of surveillance. In keeping with other trends in Malawi, health facility attendance recovered only gradually, which was reflected in reduced blood culture collection and case detection between year 2 and year 3 after the trial start date. Importantly, however, attendance and case detection recovered in the later stages, allowing us to robustly assess the durability of protection. Again, although this might result in the true incidence being underestimated, in most scenarios we would not expect that the effect of COVID-19 on attendance would cause bias or affect the estimates of vaccine efficacy, which were based on the comparison of incidences between groups, since both vaccine allocation groups should be equally affected. In the unlikely scenario that substantial typhoid cases occurred during the COVID-19 pause, then the efficacy reported herein is likely an underestimate.

In summary, this study has found high and durable efficacy of a single dose of Vi-TT across all age groups, providing crucial evidence for the probable cost-effectiveness and success of routine immunisation and mass vaccination campaigns in areas of high disease burden. Since November, 2019, Pakistan, Liberia, Zimbabwe, Samoa, and Nepal have introduced TCV through campaigns followed by routine immunisation. In Malawi, our findings informed a nationwide TCV campaign, conducted in May, 2023, for all children aged 9 months to 15 years, followed by integration of TCV into routine EPI at 9 months of age. Importantly, the previously shown high tolerability of TCV and its lack of interference with other routine vaccines^25^, ^28^, ^29^ allowed the Malawi campaign to be integrated with the delivery of the measles–rubella vaccine, oral poliovirus vaccine, and vitamin A. This integrated health programme in Malawi has come at a crucial time, when Malawi and other regional African countries are struggling with climate change, extreme weather events, and increased urbanisation; patterns which are likely to contribute to increases in enteric diseases, including typhoid fever, in the absence of urgent interventions. Importantly, the surveillance infrastructure existing in this study area for more than 10 years, and strengthened through this trial, will be used to assess the effect of the new vaccination programme.

## Data sharing

The full data set will be available on the Vivli platform (https://search.vivli.org/studyDetails/fromSearch/0bb6a051-8149-4161-bb99-d95cdc700714).

## Declaration of interests

YL, SD, LPJ, MBL, and KMN receive funding from the TyVAC grant (grant number OPP1151153). KMN is a voting member of the WHO Strategic Advisory Group of Experts on Immunization (SAGE). RSH is a UK National Institute for Health and Care Research Senior Investigator. All other authors declare no competing interests.
