# Supplementary material for: Efficacy of typhoid conjugate vaccine: final analysis of a 4-year, phase 3, randomised controlled trial in Malawian children
Source: Lancet. 2024 Feb 3;403(10425):459–68. doi: 10.1016/S0140-6736(23)02031-7 (PMC10850983; doi:10.1016/S0140-6736(23)02031-7)
Supplement: Supplementary appendix [file mmc1.pdf]

THE LANCET

Supplementary appendix

This appendix formed part of the original submission and has been peer reviewed. We post it as supplied by the authors.

Supplement to: Patel PD, Liang Y, Meiring JE, et al. Efficacy of typhoid conjugate vaccine: final analysis of a 4-year, phase 3, randomised controlled trial in Malawian children. *Lancet* 2024; published online Jan 25. https://doi.org/10.1016/S0140-6736(23)02031-7.

Supplementary Appendix

Supplementary Table 1: Additional subgroup analysis of blood culture confirmed typhoid fever and vaccine efficacy in the intention-to-treat population.

Subgroup	Number of subjects at risk	Total follow-up time (person-year)	Number of cases	Incidence rate (per 100,000 person-year)	Vaccine efficacy [95% CI]
**Sex**					
Male					
Vi-TT	7004	30071	10	33·3 [17·9, 61·8]	82·0% [64·3%,
MenA	6830	29203	54	184·9 [141·6, 241·4]	91·8%]
Female					
Vi-TT	7065	30429	14	46·0 [27·3, 77·7]	74·5% [53·6%,
MenA	7231	31017	56	180·6 [138·9, 234·6]	86·9%]
**Study site**					
Ndirande					
Vi-TT	8863	37763	15	39·7 [24·0, 65·9]	76·4% [58·0%,
MenA	8832	37509	63	168·0 [131·2, 215·0]	87·5%]
Zingwangwa					
Vi-TT	5206	22737	9	39·6 [20·6, 76·1]	80·9% [60·5%,
MenA	5229	22712	47	206·9 [155·5, 275·4]	91·8%]

CI = confidence interval; Vi-TT = Vi polysaccharide tetanus toxoid typhoid conjugate vaccine; MenA = meningococcal capsular group A conjugate vaccine.

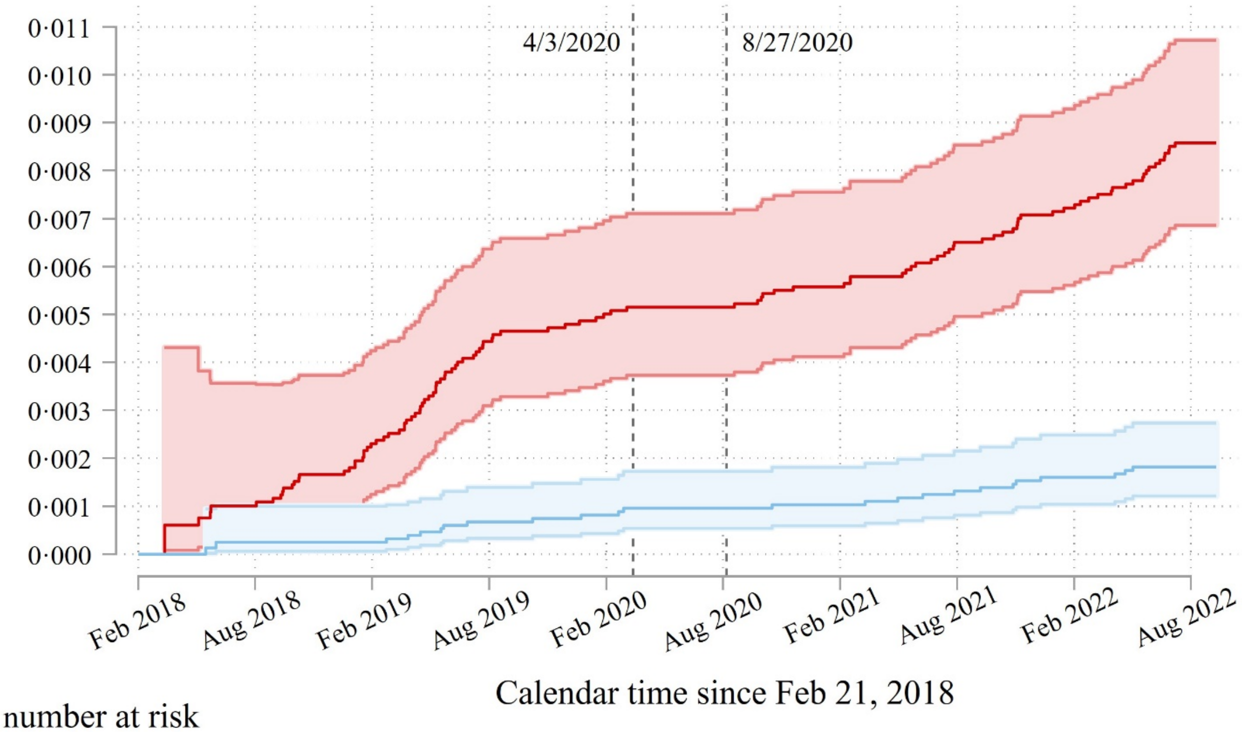

Maximum number at risk

MenA:	11993	14023	13989	13978	13974	13968	13912	13902	13883
Vi-TT:	12030	14055	14048	14045	14043	14039	13988	13983	13979

95% CI — MenA
95% CI — Vi-TT

Supplementary Figure 1: Kaplan-Meier estimates of the cumulative incidence of blood culture positive typhoid fever beginning on the first enrollment date in the intention-to-treat population by vaccine group. Dates of COVID-19 surveillance interruptions are shown with dotted vertical lines. Maximum number of participants at risk are shown below the graph.

CI = confidence interval; MenA = meningococcal capsular group A conjugate vaccine; Vi-TT = Vi polysaccharide tetanus toxoid typhoid conjugate vaccine.

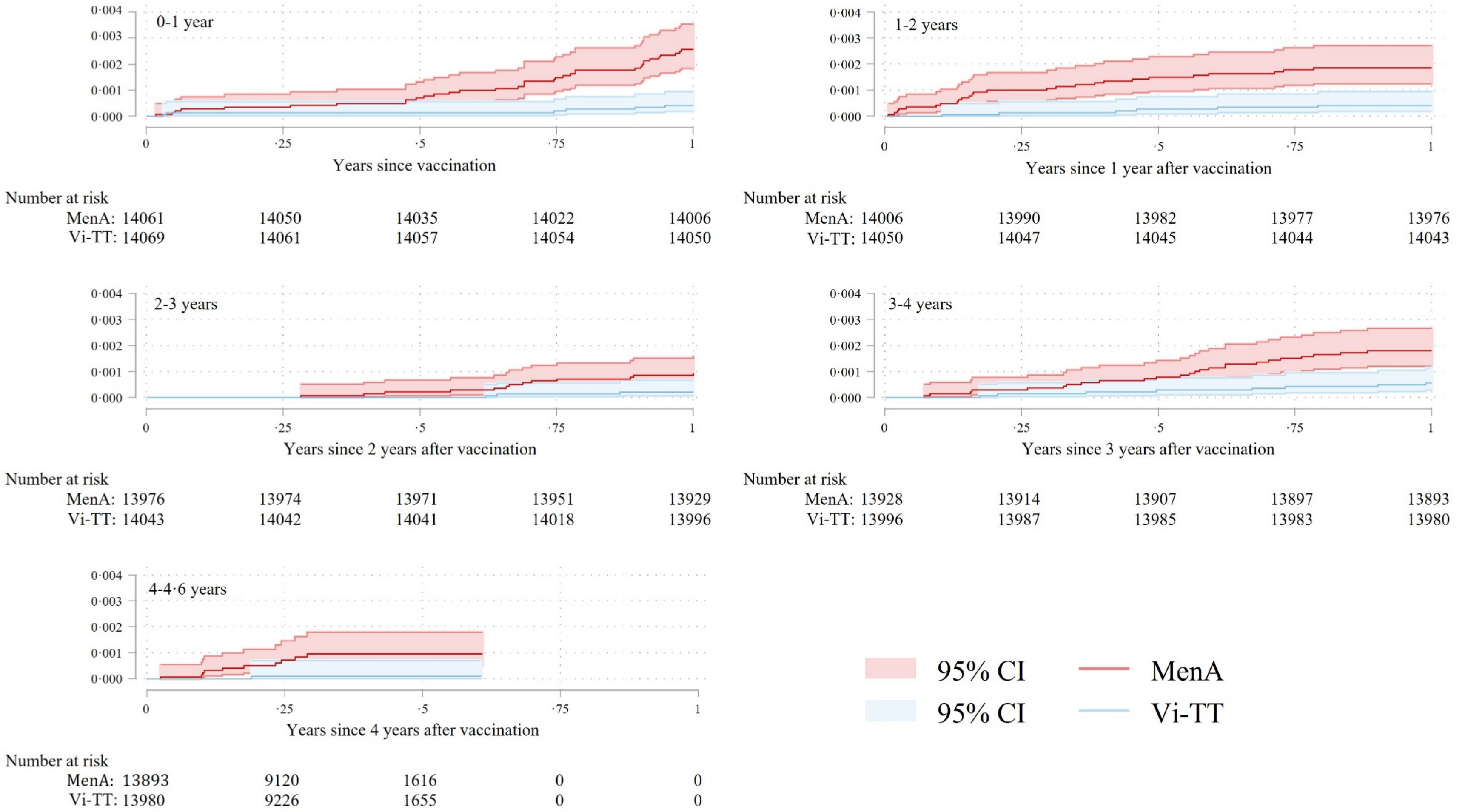

Supplementary Figure 2: Kaplan-Meier estimates of the cumulative incidence of blood culture positive typhoid fever over five time intervals (0-1 year, 1-2 years, 2-3 years, 3-4 year, 4-4.6 years) in the intention-to-treat population by vaccine group. Maximum number of participants at risk is shown below each graph. CI = confidence interval; MenA = meningococcal capsular group A conjugate vaccine; Vi-TT = Vi polysaccharide tetanus toxoid typhoid conjugate vaccine.

Vi-TT = Vi polysaccharide tetanus toxoid typhoid conjugate vaccine; MenA = meningococcal capsular group A conjugate vaccine.

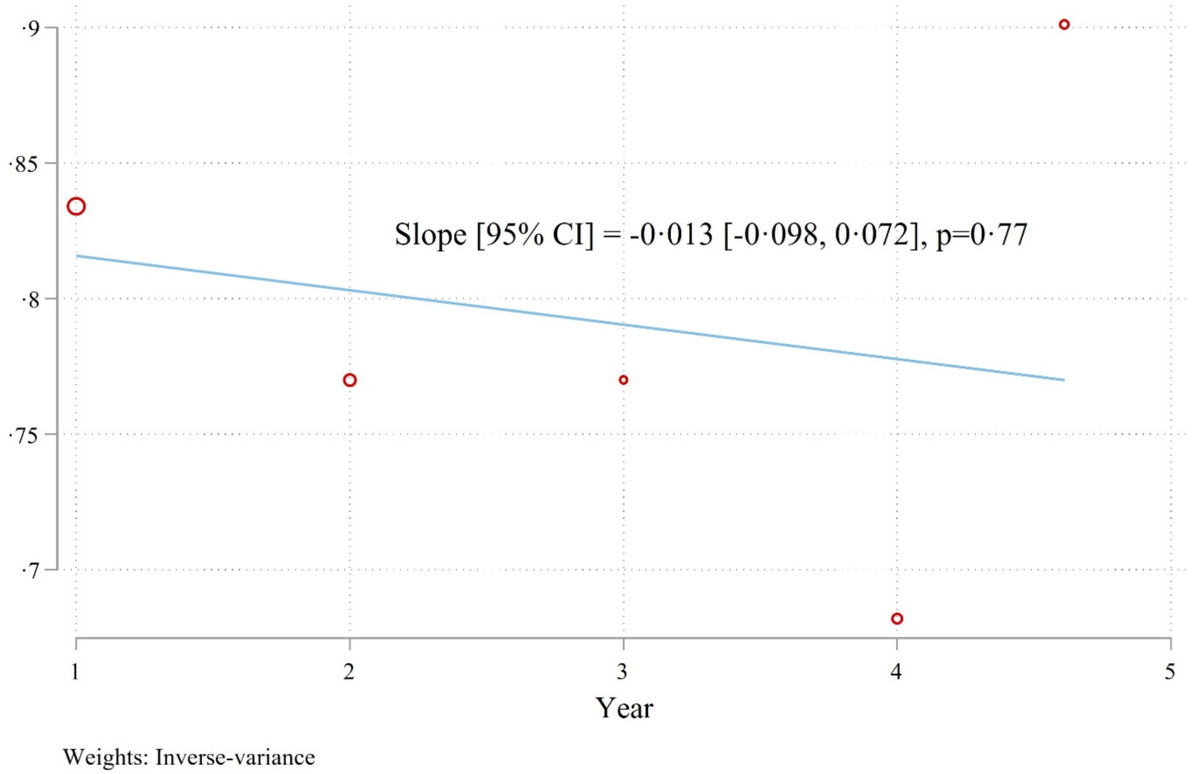

Supplementary Figure 3: Meta-regression of vaccine efficacy over time in the intention-to-treat population. Open circles represent the point estimate of efficacy at the end of each of five time periods (0-1, 1-2, 2-3, 3-4, and 4-4·61 years after vaccination).

CI = confidence interval. Size of the circle is proportional to the precision of the vaccine efficacy estimation. Larger the circle, the more precise the vaccine efficacy estimation (i.e., narrower the 95% confidence interval).

TyVAC study team

Malawi–Liverpool–Wellcome Programme

- Chrissy Banda
- David Banda
- Josephine Chilongo
- Amisa Chisale
- Mark Haward
- Harrison Msuku
- John Ndaferankhande
- Chancy Nyirongo
- Patricia Phula
- James Tamani

Blantyre Malaria Project

- Victoria Mapemba

University of Maryland School of Medicine

- Fleesie Hubbard
- Melissa Myers
- Tamar Pair
